# Electrical Properties and Reliability of AlGaN/GaN High Electron Mobility Transistor under RF Overdrive Stress at High Temperature

**DOI:** 10.3390/mi15091100

**Published:** 2024-08-30

**Authors:** Chang Liu, Yiqiang Chen, Yuhan Xie, Hongxia Liu, Zongqi Cai

**Affiliations:** 1 School of Microelectronics, Xidian University, Xi’an 710071, China; xd_liuchang@163.com; 2 China Electronic Product Reliability and Environmental Testing Research Institute, Guangzhou 511370, China

**Keywords:** gallium nitride, high electron mobility transistor, high-temperature reliability, radio frequency

## Abstract

We have investigated the electrical properties and reliability of AlGaN/GaN high electron mobility transistors (HEMT) under high-temperature RF overdrive stress. The experimental results show that the drain current and transconductance of the device decrease at 25 °C and 55 °C but do not change significantly at 85 °C before and after the stress. The decline rate of the saturation drain current, the degradation amplitude of transconductance, and the drift amplitude of threshold voltage decrease with the increase in temperature. The results of pulse *I*–*V* and low-frequency noise tests show that the current collapse is inhibited, and the trap density is reduced at higher temperatures. The Electroluminescence (EL) test shows that the luminescence characteristics of the device after RF overdrive stress are more scattered and weaker. We believe that the degradation at lower temperatures is mainly due to the influence of the hot electron effect (HEE), while the change in electrical properties at higher temperatures is due to the weakening of HEE and the improvement of the Schottky interface.

## 1. Introduction

The first-generation semiconductor devices represented by Si and Ge and the second-generation semiconductor devices represented by GaAs have been widely used in various electronic devices. Although the first- and second-generation semiconductor devices are quite advanced technologies, the application of the first two generations of semiconductor electronic devices in high temperature, high frequency, and high power is limited due to various shortcomings of the materials themselves. Since the emergence of the third-generation semiconductor devices represented by GaN, they have rapidly become the focus of the latest research in the semiconductor industry. Compared with the previous two generations, the third-generation semiconductor devices not only eliminate their shortcomings but also have more obvious advantages. The GaN semiconductor is an excellent material for high-frequency, high-temperature, and high-power devices due to its wide bandgap, very high electron mobility, high-breakdown electric field resistance, good heat resistance, and excellent radiation resistance [1,2,3,4]. In recent years, GaN-based high electron mobility transistor (HEMT) devices have become a hot research topic in electronic power components. It is expected that in future, wide bandgap semiconductors will be widely used in military radar, laser weapons, aerospace, and other important fields, such as information and communication, electric locomotives, and other electronic products [5].

However, the reliability of GaN HEMTs has been a stumbling block for their further development. In particular, the degradation effect of GaN HEMTs under RF stress is of great concern, considering that GaN HEMTs actually operate in the RF state. Previous studies have shown that RF stress can cause different degradation phenomena from DC stress [6,7,8]. Meanwhile, compared with the saturate RF stress, the RF overdrive stress often produces more serious degradation to the device since the excess input power is converted into heat, which further increases the junction temperature of the device. Many previous studies have investigated the degradation effects of GaN HEMTs under RF overdrive stress [9,10,11,12,13], but the experiments were usually conducted at room temperature or a fixed temperature [14,15], and the correlation between the degradation effects and temperature was not studied to further explore the role of temperature in device degradation. In this paper, the short plate will be repaired, and the influence rule of temperature on device degradation will be studied by carrying out RF overdrive experiments under different ambient temperatures.

This paper is arranged as follows. In the second section, we briefly introduce the construction of the experimental platform. In the third section, we discuss the DC, pulse *I–V*, low-frequency noise, and EL characteristics of the device before and after the stress. Then, we propose an explanation of the degradation mechanism. The last part summarizes this paper.

## 2. Materials and Methods

**Figure 1:**
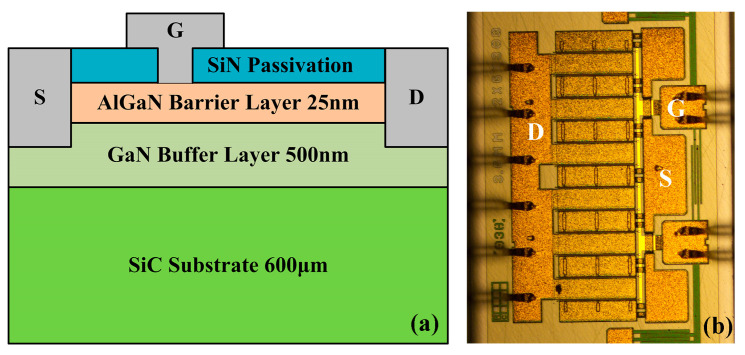
(**a**) Schematic of cross-section and (**b**) surface morphology of AlGaN/GaN HEMTs.

Figure 1 is schematic diagram and surface morphology of the device, respectively. It comprises a 600 μm SiC substrate, a 500 nm GaN buffer layer, a 25 nm AlGaN barrier layer, and a SiN passivation layer. The device has a gate length of 0.35 μm and a gate width of 3.6 mm. Its operating frequency ranges from 1.1 GHz to 1.2 GHz.

**Figure 2:**
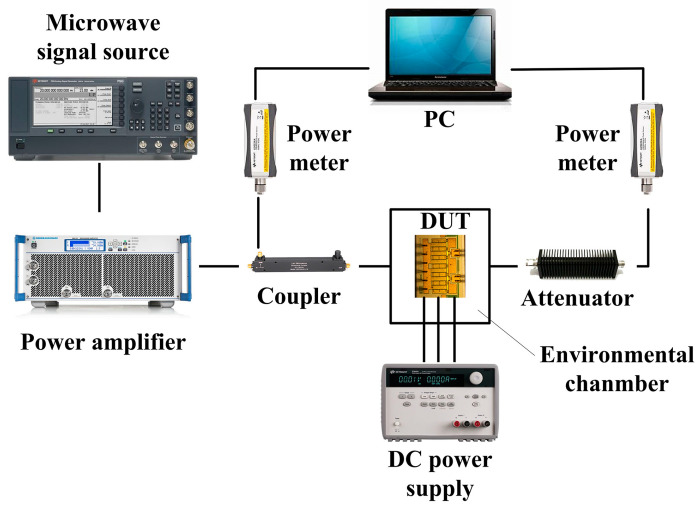
Schematic diagram of RF overdrive stress experimental platform.

Figure 2 shows the composition of the experimental platform. The experimental platform consists of an environmental chamber manufactured by GWS (Guangzhou, China), DUT, signal source manufactured by Keysight (Santa Rosa, CA, USA), power amplifier manufactured by R&S (Munich, Germany), directional coupler, DC power supply manufactured by Keysight (Santa Rosa, CA, USA), attenuator, and two load-type power meters manufactured by Keysight (Santa Rosa, CA, USA). The DC power supply provides bias conditions for normal operation of the device, and the environmental chamber provides a constant ambient temperature for the DUT. The signal source provides the input signal and reaches the signal input end of the device through the power amplifier and the coupler. The purpose of setting the power meter is to monitor the input and output power of the device in the whole experiment process, and the attenuator at the front of the power meter is to prevent the power meter from being damaged due to excessive power.

During the experiment, the device was placed in the environmental chamber, and the experiment temperatures were 25 °C, 55 °C, and 85 °C, respectively. The temperature is stable during the RF overdrive stress and only returns to room temperature when the experiment is over. The DC bias of the device was fixed as *V*_ds_ = 28 V and *V*_gs_ = −2.5 V and continuous wave (CW) signal with frequency of 1.15 GHz input into the device. The input power was 5 dB higher than the saturated input power. All experiments lasted 100 h at different ambient temperatures. The electrical, low-frequency noise and EL characteristics of the device were measured before the stress at room temperature, and these characteristics were measured again after removing the RF overdrive stress and waiting for the temperature to return to room temperature. For output characteristic measurements, the *V*_ds_ scans from 0 V to 10 V, and the *V*_gs_ selects 7 bias points between −3.25 V and −1.75 V. When measuring the transfer curve, the *V*_ds_ is fixed at 0.1 V, and the *V*_gs_ scans from −5 V to 0 V. The *V*_gd_ or *V*_gs_ scan from −15 V to −2 V in the gate leakage current characteristic measurement. When measuring pulse *I*–*V* characteristics, the static bias points were selected as (*V*_gsq_, *V*_dsq_)= (0 V, 0 V), (−8 V, 0 V), and (−8 V, 10 V). The frequency range of LFN measurement was from 1 Hz to 10 kHz, and the *V*_ds_ were fixed at 0.1 V. Keysight B1500A manufactured by Keysight (Santa Rosa, CA, USA) was used to measure the electrical characteristics, and Keysight E4727A manufactured by Keysight (Santa Rosa, CA, USA) was used to measure the low-frequency noise characteristics.

## 3. Results

### 3.1. DC Characteristics

**Figure 3:**
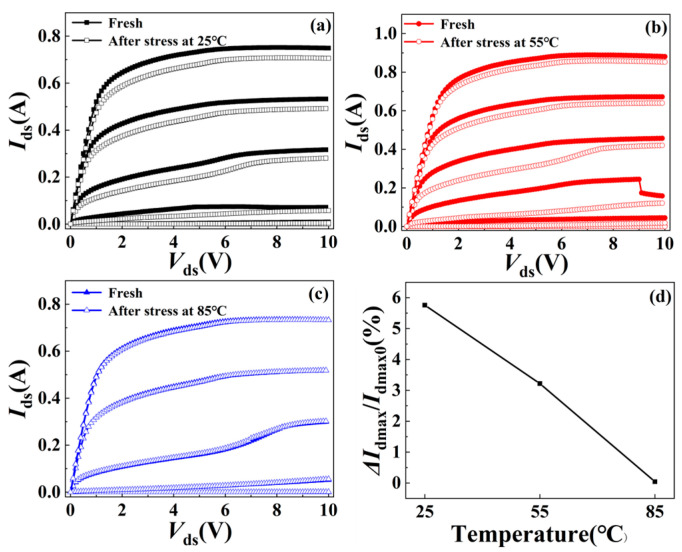
Output characteristics of the device before and after RF overdrive stress at (**a**) 25 °C, (**b**) 55 °C, and (**c**) 85 °C with *V*_gs_ ranging from −3.25 V to −1.75 V at a step of 0.25 V. (**d**) Change rate of saturation drain current *I*_dmax_ (measured at *V*_ds_ = 10 V, *V*_gs_ = −1.75 V) at different ambient temperatures.

Figure 3a–c show the output characteristics (*I*_ds_ − *V*_ds_) with *V*_gs_ ranging from −3.25 V to −1.75 V at a step of 0.25 V before and after the stress at different ambient temperatures. It should be noted that the sudden fluctuations in the output characteristics of the fresh device, as shown in Figure 3b, are caused by the self-excited oscillation. When the ambient temperature is 25 °C, the saturation drain current *I*_dmax_ (*V*_ds_ = 10 V, *V*_gs_ = −1.75 V) of the device decreased from 748 mA to 705 mA. At 55 °C, the *I*_dmax_ decreases from 880 mA to 852 mA. However, *I*_dmax_ does not change significantly and remains at 732 mA at 85 °C. It can be seen that the deterioration of device output current grows lower as the temperature rises. The relationship between the change rate of *I*_dmax_ and the ambient temperatures of the device is shown in Figure 3d. The change rate of *I*_dmax_ at 25 °C, 55 °C, and 85 °C is 5.76%, 3.22%, and 0.03%, respectively, indicating that the degradation effect is negatively correlated with temperature.

**Figure 4:**
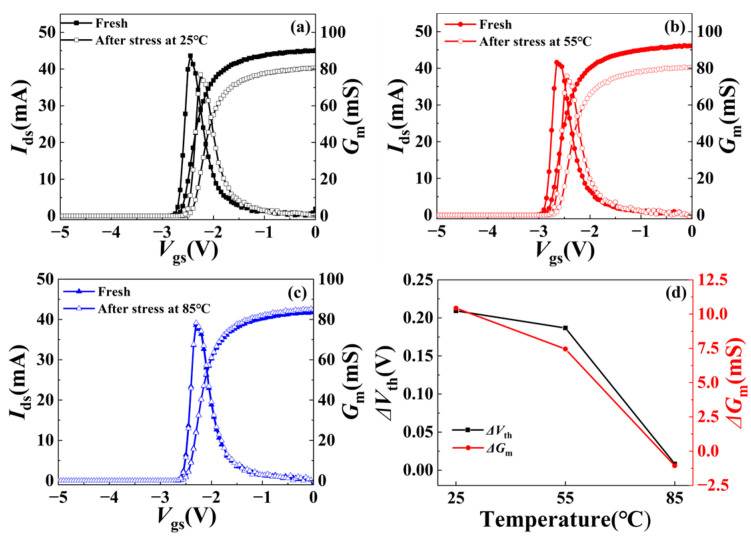
Transfer characteristics of the device before and after RF overdrive stress at (**a**) 25 °C, (**b**) 55 °C, and (**c**) 85 °C with *V*_ds_ = 0.1 V. (**d**) The variation of threshold voltage *V*_th_ and maximum transconductance *G*_mmax_ at different ambient temperatures.

Figure 4a–c show the transfer characteristics (*I*_ds_ − *V*_gs_) with *V*_ds_ of 0.1 V before and after the stress at different ambient temperatures. When RF overdrive stress is applied at 25 °C and 55 °C, the threshold voltage (*V*_th_) of the device drifts forward slightly, and the maximum transconductance (*G*_mmax_) decreases, while *V*_th_ and *G*_mmax_ are almost unchanged at 85 °C. Figure 4d illustrates the relationship between the change in *V*_th_ and *G*_mmax_ and ambient temperature. The *G*_mmax_ decreased from 87 mS to 76 mS, with a decrease rate of 12% at 25 °C, while it remained 77 mS at 85 °C. The change in threshold voltage also follows the same rule, which is consistent with the drain current shown in Figure 3d. These phenomena may indicate that trapping behavior has occurred at lower temperatures [16,17].

**Figure 5:**
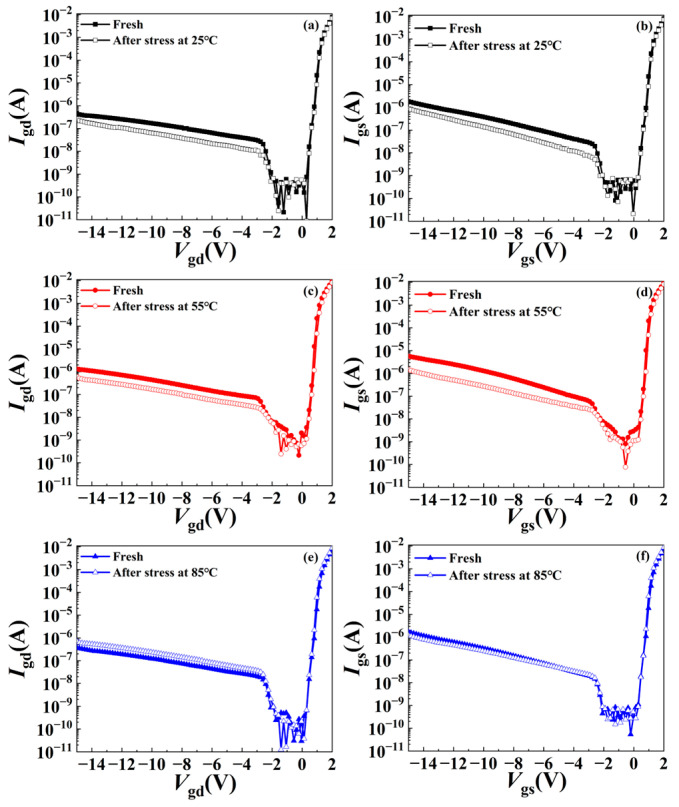
Gate leakage current characteristics at 25 °C, 55 °C, and 85 °C. (**a**,**c**,**e**) are gate–drain leakage current. (**b**,**d**,**f**) are gate–source leakage current.

The gate leakage current characteristics before and after the stress have also been measured to demonstrate the effect of stress on the Schottky contact, as shown in Figure 5. It can be seen from the figure that both the gate–drain current and gate–source current under the negative bias decrease significantly at 25 °C and 55 °C, which may be due to the decrease in the electric field near the gate caused by the significant surface trapping effect at lower temperatures [18,19]. However, both the gate–drain current and gate–source leakage current change little before and after the stress at 85 °C, which indicates that the trapping effect is weakened at higher temperatures, which is also consistent with the change in output and transfer characteristics. This will be further demonstrated by pulse *I*–*V* and low-frequency noise tests.

### 3.2. Pulse I–V Properties

**Figure 6:**
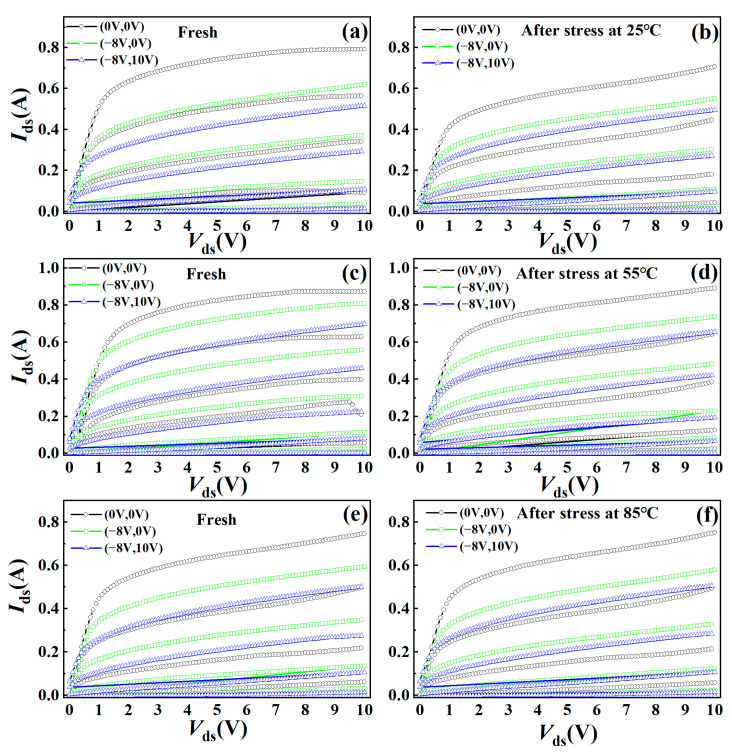
Pulse *I*–*V* characteristics at 25 °C, 55 °C, and 85 °C. (**a**,**c**,**e**) are fresh devices. (**b**,**d**,**f**) are the devices after stress. The static bias points were selected as (*V*_gsq_, *V*_dsq_) = (0 V, 0 V), (−8 V, 0 V) and (−8 V, 10 V).

A pulse *I*–*V* test was carried out before and after the stress in order to test the current collapse effect of the device. The static bias points *V*_gsq_ and *V*_dsq_ were selected at (0 V, 0 V), (−8 V, 0 V), (−8 V, 10 V) in the test, as shown in Figure 6. 

Table 1 lists the current collapse ratio before and after stress at different ambient temperatures. The current collapse rate is defined as the decline rate of *I*_ds_ when the static bias point is (−8 V, 0 V) and (−8 V, 10 V) compared to that when the static bias point is (0 V, 0 V). The reference bias point is *V*_ds_ = 10 V, *V*_gs_ = −1.75 V. After RF overdrive stress, the current collapse rate of the device generally increases, but the current collapse rate at 85 °C is less than that at 25 °C, which means the current collapse effect of the device can be alleviated with the increase in temperature. On the other hand, it also indicates that fewer electrons are trapped in the barrier layers or the surface at higher temperatures.

### 3.3. Low-Frequency Noise

**Figure 7:**
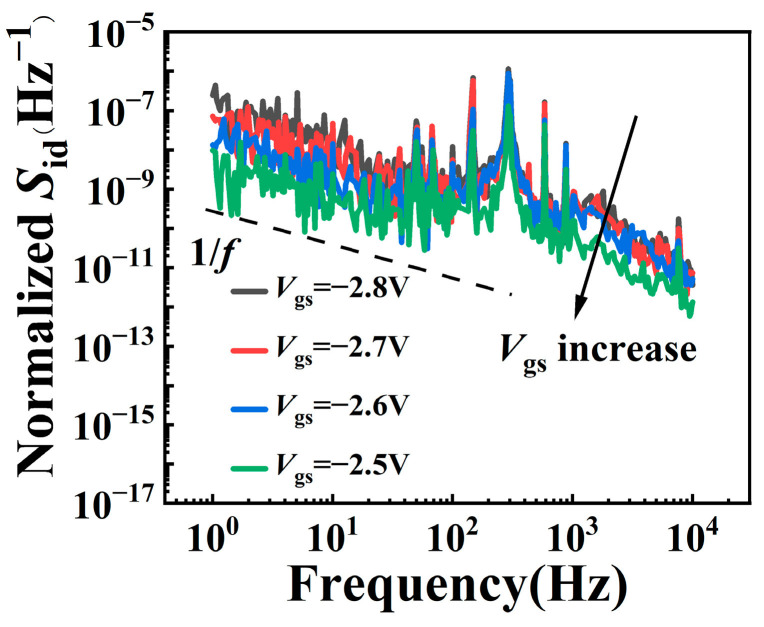
Typical normalized *S*_id_ versus frequency with *V*_ds_ = 0.1 V and *V*_gs_ ranging from −2.8 V to −2.5 V at a step of 0.1 V.

In order to further analyze the trap behavior inside the device caused by RF overdrive stress at different ambient temperatures, the low-frequency noise test was carried out. The drain voltage was biased at 0.1 V, and the frequency ranged from 1 Hz to 10 kHz. The normalized *S*_id_ (*S*_id_/*I*_ds_^2^) is shown in Figure 7. From the figure, it can be seen that the normalized *S*_id_ decreases with the increase in gate voltage. The curve trend conforms to the law of 1/*f* at a low frequency.

**Figure 8:**
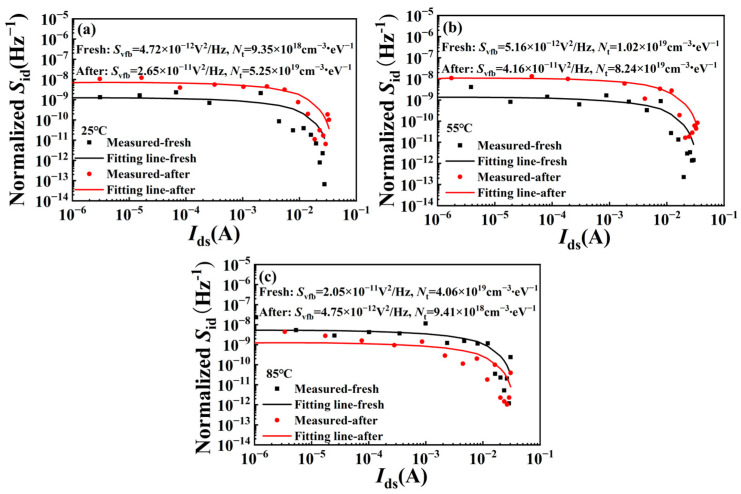
Typical normalized *S*_id_ versus *I*_ds_ at (**a**) 25 °C, (**b**) 55 °C, and (**c**) 85 °C before and after RF overdrive stress.

To analyze the trap density, we utilize the classical Δ*n* − Δ*μ* model [20,21]; the normalized *S*_id_ can be expressed as

(1) $$\frac{S_{\mathrm{id}}}{I_{\mathrm{ds}}^{2}}={\left(\frac{G_{m}}{I_{\mathrm{ds}}}\right)}^{2}S_{\mathrm{vfb}},$$

where *S*_vfb_ is flat band voltage spectral density. Based on (1), we can determine the relationship between normalized *S*_id_ and *I*_ds_ and calculate the value of *S*_vfb_ through fitting, as shown in Figure 8. The relationship between *S*_vfb_ and trap density *N*_t_ is as follows [22,23,24]:

(2) $$S_{\mathrm{vfb}}=\frac{q^{2}kT\lambda N_{t}}{WLfC_{b}^{2}},$$

Where *λ* = 0.5 nm is conduction band alignment, *W* is the gate width, *L* is the gate length, and *C_b_* is the AlGaN unit barrier capacitance. The trap density *N*_t_ values before and after the stress at different temperatures can be derived by (2). At 25 °C, *N*_t_ increased from 9.35 × 10^18^ cm^−3^∙eV^−1^ to 5.25×10^19^ cm^−3^∙eV^−1^, and at 55 °C, *N*_t_ increased from 1.02×10^19^ cm^−3^ eV^−1^ to 8.24 × 10^19^ cm^−3^∙eV^−1^, while at 85 °C, *N*_t_ decreased from 4.16 × 10^19^ cm^−3^ eV^−1^ to 9.41 × 10^18^ cm^−3^ eV^−1^. In other words, the trap density of the device increases after RF overdrive stress at 25 °C and 55 °C but decreases after the stress at 85 °C. Obviously, the increase in trap density at lower temperatures is a good validation of the degradation seen in electrical tests, proving that the hot electron effect caused by RF overdrive stress creates a new trap inside the device with more trapped electrons [14,19]. The decrease in trap density at 85 °C may be attributed to the improvement of Schottky contact properties at high temperatures [25,26].

### 3.4. Electroluminescence Properties

**Figure 9:**
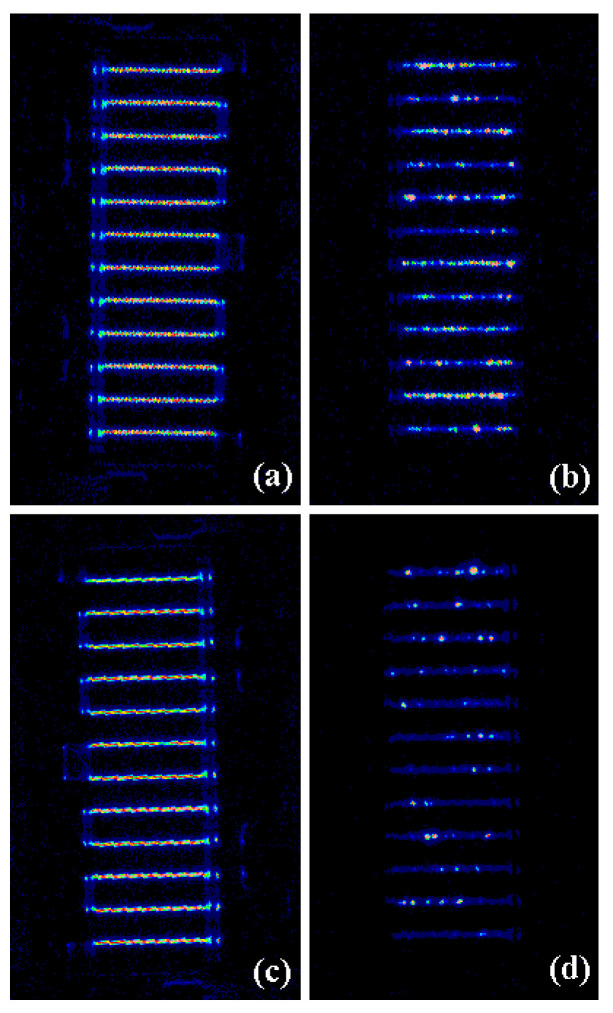
EL images of the AlGaN/GaN HEMTs. (**a**) Fresh device under DC bias, (**b**) fresh device under RF overdrive bias, (**c**) the device under DC bias after RF overdrive stress at 25 °C, (**d**) the device under RF overdrive bias after RF overdrive stress at 25 °C.

Electroluminescence (EL) is an effective means to detect hot electrons in GaN devices, so the EL test was performed on the devices before and after the stress at 25 °C in order to confirm whether the hot electron effect caused the degradation at lower temperatures. Figure 9a,b show the EL image of the device under DC bias and RF overdrive bias before the stress at 25 °C. Figure 9c,d show the EL image of the device under DC bias and RF overdrive bias after the stress at 25 °C. The DC bias is *V*_ds_ = 28 V and *V*_gs_ = −2.5 V, while the overdrive bias is *V*_ds_ = 28 V, *V*_gs_ = −2.5 V, and *P*_in_ = 38 dBm. The Emission Microscope we used here can detect light signals in the wavelength range of 900 nm to 1650 nm.

When the device is biased at DC, it can be seen that the overall luminous phenomenon is relatively uniform (see Figure 9a,c). However, when the device is applied to RF overdrive bias, the luminescence is not uniform any more. Compared with the EL image under RF overdrive bias before the stress (Figure 9b), the luminescence characteristics after the stress (Figure 9d) are more scattered and weaker, indicating that the device has undergone obvious degradation. Under RF stress, the high field drives the charge carriers in the channel to accelerate, gaining enough energy to become hot electrons. After escaping from the potential well, some hot electrons generate new permanent defects. Other hot electrons can be captured by some inherent traps introduced in the fabrication process of the device, resulting in device degradation [27]. 

## 4. Discussion

**Figure 10:**
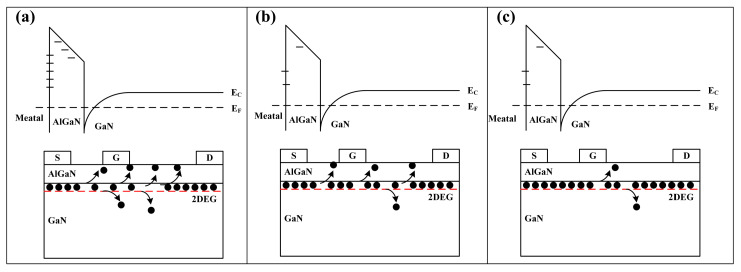
The schematic of the degradation mechanism at (**a**) 25 °C, (**b**) 55 °C, and (**c**) 85 °C.

In order to better analyze the mechanism, we summarized the variation of the device characteristics in Table 2. The direction of the arrows in the table indicates whether the parameter increases or decreases after the RF overdrive stress, and the number of arrows indicates the magnitude of the change. A schematic of the degradation mechanism is shown in Figure 10. 

In brief, the degradation mechanism of devices under RF overdrive stress at lower temperatures (25 °C and 55 °C) is related to the hot electrons effect [9,11,20], while at higher temperatures (85 °C), the hot electron effect is weakened and the Schottky interface is improved, resulting in little change in most parameters.

The specific analysis is as follows. First of all, the change in *I*_dmax_ can be explained by the saturation current formula [28]:

(3) $$I=qn_{s}Wv(x)$$

where *q* is electronic charge, *W* is channel width, *ν*(*x*) is carrier drift speed, and *n*_s_ is channel carrier density. Obviously, the parameters that can change the saturation current are *ν*(*x*) and *n_s_*, where *ν*(*x*) decreases as the temperature rises. Considering that the measurements before and after the experiment are carried out at room temperature, *ν*(*x*) should be constant, and only *n*_s_ affects the saturation current. At 25 °C, the hot electron effect is the most obvious. The electrons in the channel gain enough energy and escape from the potential well. These electrons can be captured by the trap of the buffer layer, barrier layer, or surface, which is introduced during the manufacturing process at the current state of the art [29]. The hot electron effect can also produce new traps that can capture electrons further [12,14,18], so 2DEG can be partially depleted, resulting in a drop in output current, as shown in Figure 10a. When the temperature rises to 55 °C, the hot electron effect is slightly suppressed, and the *n*_s_ decline range decreases, as shown in Figure 10b, so the drain current shows a decline, but the decline amplitude decreases. When the temperature reaches 85 °C, as shown in Figure 10c, lattice vibration becomes more intense, and electrons in the channel are more likely to collide with lattice atoms. The average free path of electrons decreases, and the maximum kinetic energy of carriers decreases according to phonon scattering [30], which results in a decrease in the number of hot electrons in the channel [8,31,32]. At the same time, according to the LFN test results, the trap density in the device has decreased, so the *I*_dmax_ after the stress at 85 °C has basically not decreased.

The changes in *V*_th_, *G*_mmax_, and current collapse rate can be explained by the virtual gate effect. As mentioned above, at lower temperatures, the hot electron effect will create new traps (confirmed by LFN as shown in Figure 8a,b), resulting in a more obvious trapping effect. Some electrons will be captured by the trap below the gate, forming a virtual gate, which will produce an additional modulation effect on the gate voltage, resulting in *V*_th_ forward drift, *G*_mmax_ decrease, and current collapse effect enhancement. However, when the temperature rises, the hot electron effect is suppressed, and the trapped electrons below the gate are reduced. Meanwhile, high temperature may also improve the Schottky contact interface and reduce the trap density [33], as shown in Figure 8c, and further weaken the virtual gate effect, resulting in little change in *V*_th_, *G*_mmax,_ and current collapse rate at 85 °C.

However, the change in gate leakage current mainly comes from the change in the tunneling mechanism. As can be seen from Figure 5, the gate leakage current mainly changes significantly under negative bias voltage, so the main leakage current mechanism includes FN tunneling and FP tunneling, where FN tunneling is related to the electric field while FP tunneling is related to the trap density. At lower temperatures, electrons are trapped near the gate, which reduces the electric field, leading to the weakening of the FN tunneling mechanism, so the gate leakage current decreases. At 85 °C, the reduced traps will also obviously weaken the FP emission mechanism. At the same time, considering the reduction of traps below the gate, the electric field near the gate will increase, and the FN tunneling will be enhanced, so under the combined action, the gate leakage current at 85 °C does not change much.

## 5. Conclusions

In this paper, room- and high-temperature RF overdrive stress tests were carried out on AlGaN/GaN HEMTs to investigate the electrical properties and reliability of the devices. As the temperature increased from 25 °C to 55 °C to 85 °C, the drain current of the device decreased by 5.76%, 3.22%, and 0.03%, respectively. The maximum transconductance decreased from 87 mS to 76 mS at 25 °C and decreased from 83 mS to 75 mS at 55 °C but remained at 77 mS at 85 °C. Low-frequency noise tests and pulse *I–V* tests showed that the trap density increases obviously under RF overdrive stress at lower temperatures, but high temperatures suppress this phenomenon. The Electroluminescence (EL) test proved the main mechanism of device degradation at lower temperatures is the hot electron effect. We believe that the mechanism of device degradation at lower temperatures (25 °C and 55 °C) is mainly the hot electron effect, while at high temperatures (85 °C), the hot electron effect is suppressed and the Schottky interface is improved, resulting in the reduction of device degradation. The results found in this paper confirm that AlGaN/GaN HEMTs have a good tolerance to RF overdrive stress in high-temperature environments, but it is still necessary to avoid an overdrive state in practical applications. Additionally, previous studies have shown that GaN HEMTs will also degrade significantly at higher temperatures [15], and the reliability of RF stress over a wider temperature range is worthy of further study. In order to restrain the degradation caused by RF stress, the Fe-C co-doped buffer layer, the thinner barrier layer, and the optimized gate structure can be used [34,35,36]. 

## Figures and Tables

**Table 1:** Current collapse ratio at different ambient temperatures before and after the stress.

Temperature	Current Collapse Ratio (Reference Point: *V*_ds_ = 10 V, *V*_gs_ = −1.75 V)					
	(−8 V, 0 V)			(−8 V, 10 V)		
	Fresh	After	Increment	Fresh	After	Increment
25 °C	17.3%	22.0%	4.7%	29.9%	31.1%	1.2%
55 °C	8.1%	13.4%	5.3%	20.7%	23.1%	2.7%
85 °C	18.9%	20.8%	1.9%	31.4%	30.9%	−0.5%

**Table 2:** Changes in device characteristics after RF overdrive stress tests at different temperatures. “↑” means increase, “↓” means decrease, “≈” means basically unchanged, and the number of arrows indicates the magnitude of the relative change.

Characteristics	25 °C	55 °C	85 °C
*I*_dmax_	↓↓	↓	≈
*V*_th_	↑↑	↑	≈
*G*_mmax_	↓↓	↓	≈
Gate leakage current	↓	↓	≈
Current collapse ratio	↑	↑	≈
*N*_t_	↑	↑	↓

## Data Availability

The original contributions presented in the study are included in the article, further inquiries can be directed to the corresponding authors.
